# Psychometric Validation of the Malay Version of the Emotional Abuse Questionnaire (EAQ-M)

**DOI:** 10.21315/mjms-09-2024-724

**Published:** 2025-06-30

**Authors:** Muhammad Khairul Amri Yusoff, Fahisham Taib, Surini Yusoff, Nurul Jannah Ambak

**Affiliations:** Department of Paediatric, School of Medical Sciences, Health Campus, Universiti Sains Malaysia, Kelantan, Malaysia. Department of Paediatric, Hospital Pakar Universiti Sains Malaysia, Kelantan, Malaysia

**Keywords:** emotional abuse, child abuse, psychometrics, confirmatory factor analysis, validation studies

## Abstract

**Background:**

Emotional abuse is a pervasive form of maltreatment with severe mental health consequences, particularly in children. Despite its impact, emotional abuse often goes unnoticed, complicating its detection. In Malaysia, increasing awareness underscores the need for reliable and culturally adapted tools such as the Emotional Abuse Questionnaire-Malay Version (EAQ-M).

**Methods:**

This cross-sectional validation study aimed to translate and validate the EAQ into Malay. The process involved forward and backward translation, finalised by six bilingual experts, including paediatricians, certified language lecturers, and medical officers. Face validity was assessed by 10 children aged 13 to 17 years, and content validity by six experts. A total of 165 secondary school students from Kota Bharu, Kelantan, participated. Confirmatory Factor Analysis (CFA) assessed construct validity, and Cronbach’s alpha evaluated internal consistency. Test-retest reliability was determined using intraclass correlation coefficients (ICC).

**Results:**

The EAQ-M demonstrated high content validity (S-CVI/UA = 0.96) and face validity (S-FVI/UA and S-FVI/Ave = 1). CFA confirmed the retention of 29 out of 30 items, maintaining the original six-factor structure. Fit indices indicated acceptable model fit (NC = 1.555, RMSEA = 0.058, GFI = 0.819, AGFI = 0.782, CFI = 0.873, TLI = 0.857, NFI = 0.717, and SRMR = 0.081). Internal consistency was high (Cronbach’s alpha = 0.902), and test-retest reliability was strong (ICC = 0.917, 95% CI = 0.869–0.955).

**Conclusion:**

The EAQ-M, with 29 of the 30 original items retained, is a reliable and valid six-factor tool for assessing emotional abuse among Malaysian children.

## Introduction

Emotional abuse is a pervasive form of maltreatment with severe long-term mental health consequences, particularly in children. Unlike physical abuse, it is often invisible, making detection and intervention challenging. Yet, its impact can be equally detrimental (1). Neglect, emotional rejection, verbal aggression and controlling behaviours are forms of emotional abuse of children that can cause significant psychosocial damage (2).

Emotional abuse is increasingly recognised as a serious global problem. The World Health Organization estimates its prevalence to be significant, with a clear impact on adverse developmental outcomes (3). Research has shown that it affects cognitive development, self-esteem, and emotional stability, underscoring the need for comprehensive interventions that address both the family dynamic and the broader environment of the child (4, 5).

In Southeast Asia, emotional abuse is often underreported because of cultural norms, stigma, and lack of awareness. In Malaysia, despite legal protections under the Child Act 2001, it remains frequently overlooked (6, 7). Existing tools, such as the Childhood Trauma Questionnaire (CTQ), are limited in identifying specific forms of emotional abuse, as they primarily focus on general trauma and abuse (8).

The Emotional Abuse Questionnaire (EAQ), developed by Dr Vahid Momtaz using a sample of 328 Iranian students, was designed to address gaps in assessing emotional abuse. It evaluates different types of emotional abuse, including verbal aggression, emotional neglect, and controlling behaviours. The EAQ has been validated through exploratory and confirmatory factor analyses. Compared to the CTQ, the EAQ is designed to directly assess emotional abuse through six subscales: Verbal Abuse, Emotional Rejection, Over Control, Insufficient Control, Over Expectation, and Terrorising, with 30 items offer a detailed understanding of emotional abuse and its impact on children. Responses are rated on a 5-point Likert scale (0 = never to 4 = always); however, the original author did not specify a cut-off score for severity of emotional abuse (9).

Interestingly, a 2021 case report titled “Pattern of Parental Emotional Abuse Among Adolescent Nursing School Students” conducted in Egypt applied the EAQ and introduced a scoring interpretation. In this study, the Likert scale was scored as follows: 0 = never, 1 = almost never, 2 = sometimes, 3 = often, and 4 = always. The total score (maximum of 120) was categorised into three levels of abuse: not abused (≤ 30), sometimes/often abused (31–90), and always abused (≥ 90). The study found that two-thirds of participants had experienced emotional abuse. This highlights the potential of the EAQ to classify the severity of emotional abuse in both research and clinical settings (10).

Our study focused on analysing the EAQ’s factor structure, refining latent variables, and validating their relevance in the Malay context rather than assessing the prevalence of emotional abuse. The EAQ-M targets adolescents aged 13 to 17 years due to their heightened vulnerability during key developmental years.

## Methods

### Study Design, Sampling and Data Collection

**Figure 1:**
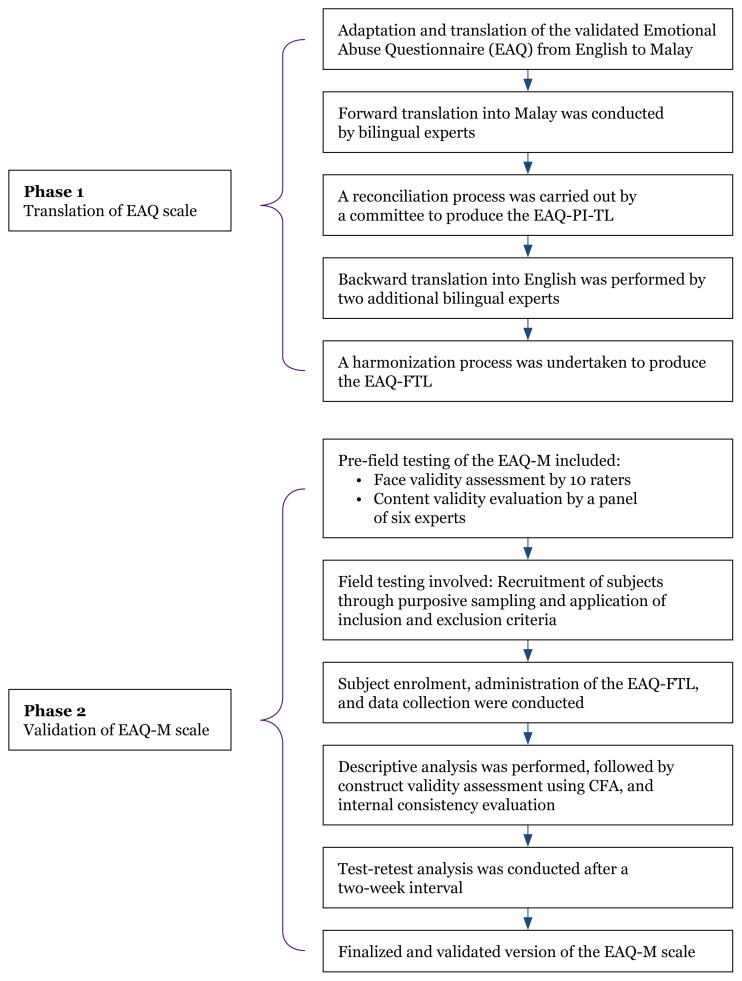
Study flowchart

This cross-sectional validation study was conducted in two phases: translation and validation (Figure 1) (11–13).

### Participant Recruitment

We recruited 165 students from a public secondary school in Kota Bharu, Kelantan, Malaysia. The recruitment process began with briefings to the school authorities about the study’s purpose, protocol, ethical considerations, and the importance of confidentiality and voluntary participation. The selected teachers and school counsellors were subsequently trained in research procedures to assist with participant recruitment. Parental consent and participants’ assent were obtained before the study commenced. After discussions with school authorities, students in Form 5 who were preparing for major exams were excluded to avoid disrupting their academic schedules. The questionnaire was administered via Google Forms, which enabled efficient data collection while maintaining anonymity through unique coding. Data collection was conducted in two rounds over a two-week period. Twenty-nine voluntary participants completed the same questionnaire twice at a two-week interval. Each session lasted approximately 30 min, with on-site researchers available to address any concerns.

### Ethical Considerations

Permission to translate and validate the EAQ was granted by its developer, Dr. Vahid Momtaz. The study protocol was approved by the Universiti Sains Malaysia Ethics Committee (USM/JEPem/KK/23020171). Parental consent and participant assent were obtained following a detailed briefing. Participation was voluntary, with the right to withdraw at any time. Confidentiality was ensured through unique coding, and data were securely stored.

### Study Phases

#### Phase 1: Translation Process of EAQ

The translation of the EAQ followed three steps: forward translation, backward translation, and harmonisation. First, two bilingual experts, a paediatric medical officer and a certified Malay language lecturer, independently translated the original English version (EAQ-SL) to Malay (EAQ-TL). The translations were then reconciled into a single version (EAQ-PI-TL) after resolving discrepancies. Next, two different translators, a general paediatrician and a certified English language lecturer, performed a backward translation of the Malay version into English. A six-member committee, including the researchers and translators, reviewed the process. The backwards-translated and original versions were compared to identify and resolve ambiguities and discrepancies. A consensus was reached to produce the pre-final version of the EAQ (EAQ-FTL), which after harmonisation became the EAQ-M (Appendix 1). This version was used for content validity, face validity, construct validity, and subsequent psychometric testing. The language lecturers involved were certified and specialised in both Malay and English teaching at the university level, which ensured linguistic accuracy and proficiency.

#### Phase 2: Validation and Reliability of the EAQ-M

##### Content validity

The content validity of the EAQ-M was evaluated by a panel of six experts: a paediatric medical officer, a psychiatry medical officer, a general paediatrician, a child psychologist, and two hospital counsellors chosen for their experience with child abuse cases. Each expert rated the relevance of the item on a 4-point scale (1 = not relevant, 4 = highly relevant). The Content Validity Index (CVI) was calculated manually by averaging their scores to ensure all items accurately represented emotional abuse and were appropriate for the target population (14).

##### Face validity

Face validity testing of the EAQ-M involved 10 adolescents aged 13 to 17 years with prior parental consent. Participants rated the clarity and comprehensibility of the items on a 4-point scale (1 = not clear, 4 = very clear). Both participants and their parents received a full briefing on the purpose and procedures of the study. The Face Validity Index (FVI) was calculated by averaging these ratings. Unlike the online field study, this phase used paper-based questionnaires to ensure that participants fully understood the questions in a controlled, face-to-face setting. This approach allowed for immediate clarification, especially given the scale’s sensitive nature (15).

##### Construct validity of EAQ-M

A field study was conducted at a secondary school in Kota Bharu, Kelantan, involving participants aged 13 to 17 years. A total of 165 participants were selected with the assistance of selected teachers and school counsellors, based on the following inclusion criteria: voluntary participation, ability to understand, speak, and write in Malay, parental consent and participant assent. Participants were excluded if they had any history of psychiatric disorders, substance abuse, or an inability to understand Malay, as identified through school counselling records.

#### Reliability and Stability Testing

The reliability of the EAQ-M was assessed using Cronbach’s alpha to measure internal consistency. Stability over time was evaluated using the ICC. Twenty-nine participants repeated the questionnaire after two weeks.

### Data Analysis

Data analysis was performed using IBM SPSS version 27. Descriptive statistics were used to summarise the socio-demographic characteristics of the sample. Numerical data are presented as means (standard deviations), while categorical data are expressed as frequencies (percentages). The content and face validity scores were manually calculated in Microsoft Excel using an index formula, with a recommended cut-off score of 0.80 for both.

The construct validity of the EAQ-M was determined using Confirmatory Factor Analysis (CFA) with IBM SPSS AMOS version 24.0 to validate the six-dimensional structure of the EAQ-M and evaluate its alignment with the original model. Based on a sample-to-variable ratio (SVR) of 5:1, for the 30 items in the EAQ-M scale, a minimum of 165 samples was required, accounting for a 10% dropout rate (16). Factor loadings below 0.30 were removed, with deletions limited to 20% to preserve the scale’s integrity. Model fit was assessed using several fit indices with the provided reference values and thresholds. Convergent and discriminant validities were evaluated.

The Kaiser-Meyer-Olkin (KMO) measure and Bartlett’s test of sphericity were used to assess sample adequacy, with values exceeding 0.6. Furthermore, Bartlett’s test of sphericity was considered significant if the *P*-value was less than 0.05 (17). Convergent validity, which ensures that items are strongly correlated within their constructs, was assessed through the Average Variance Extracted (AVE > 0.5) and Composite Reliability (CR > 0.6) (18). Discriminant validity was established by comparing the square root of the AVE and inter-factor correlation, ensuring the distinctiveness of the factors (19).

The reliability of the EAQ-M was evaluated using Cronbach’s alpha coefficients for internal consistency and ICC for test-retest stability. Items were considered to have a high internal consistency if the total alpha value was ≥ 0.60 (20), with values between 0.50 and 0.69 deemed acceptable for the newly developed scales and ≥ 0.70 preferred for established scales (21). Test-retest reliability was assessed with 29 participants who completed the EAQ-M twice at a two-week interval. Stability testing was conducted using ICC, which measures the consistency of the scale. ICC estimates and their 95% confidence intervals were calculated based on a single rating, absolute agreement and a two-way mixed model. The values were interpreted as follows: < 0.50 (poor reliability), 0.50–0.75 (moderate), 0.75–0.90 (good) and > 0.90 (excellent) (22, 23).

## Results

### Translation, Content Validation, and Face Validation of EAQ-M

The translation of the EAQ-M prioritised preserving the meaning and cultural relevance of each item. Following harmonisation among bilingual experts, minor adjustments were made to improve sentence structure and grammar for clarity and cultural appropriateness. Content validity, achieving a Scale-Content Validity Index Average (S-CVI/Ave) of 0.99 and a Scale-Content Validity Index based on Universal Agreement (S-CVI/UA) of 0.96, indicated excellent agreement on item relevance (Appendix 2). Face validity confirmed that all items were clear and easy to understand, as reflected by both S-FVI/Ave and S-FVI/UA values of 1.0, exceeding the 0.8 threshold, demonstrating the questionnaire’s clarity and suitability for Malay-speaking adolescents (Appendix 3).

### Psychometric Testing of the EAQ-M Scale

#### Characteristics of the Study Participants

This study included 165 secondary school students from Kota Bharu, Kelantan (mean age = 15.00, SD = 0.85). The sample comprised 36.4% males (*n* = 60) and 63.6% females (n = 105), all of whom were Malay. The majority of the participants lived in urban areas (90.9%, n = 150), and the median household income was RM 2,350 (IQR: RM 1,000–5,000) (Table 1).

### Construct Validity

#### Confirmatory Factor Analysis

The KMO value was 0.84, and Bartlett’s test of sphericity was significant (χ^2^ = 1,894.4, *P* = 0.01). The initial CFA model based on the original six-dimensional structure, showed an acceptable fit with a normed chi-square (NC) of 1.618 and a root mean square error of approximation (RMSEA) of 0.061. Other indices, including the goodness-of-fit index (GFI = 0.806), adjusted goodness-of-fit index (AGFI = 0.769), comparative fit index (CFI = 0.849), Tucker-Lewis index (TLI = 0.832), and normed fit index (NFI = 0.689) were close to 0.9, suggesting an acceptable fit. Factor loadings for all items range from 0.3 to 0.89; however, item F1 had a low factor loading (0.30). Consequently, a revised model (Model 2) was specified by removing item F1, which slightly improved the overall model fit. The fit indices for Model 2 remained within acceptable limits.

**Figure 2:**
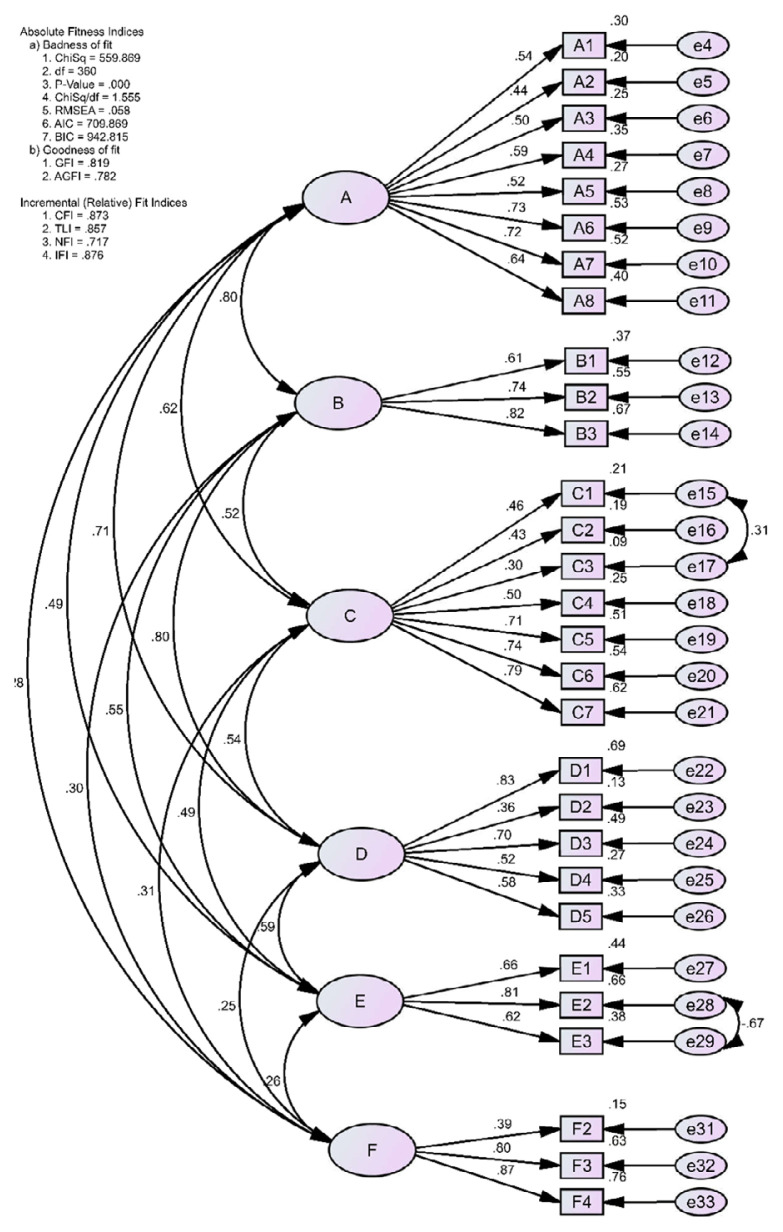
Final model (Model 4) showing the relationships between subscales: A = Verbal Abuse, B = Emotional Rejection, C = Overcontrol, D = Insufficient Control, E = Over Expectation, F = Terrorising, NC = 1.555, RMSEA = 0.058, GFI = 0.819, AGFI = 0.782, CFI = 0.873, TLI = 0.857, NFI = 0.717, SRMR = 0.081

Further respecification of Model 2 (Model 3), which included correlating the error terms for items C1 and C3, led to an improved model fit, with acceptable values for GFI (0.815), AGFI (0.777), CFI (0.863), TLI (0.846), and NFI (0.709). A similar approach was applied to Model 3 by correlating the error terms for items E2 and E3, resulting in the final Model 4, which also demonstrated an acceptable model fit. The final CFA results after specification are summarised in Table 2, with graphical representations of the models presented in Figure 2. The CFA showed that the EAQ-M retained 29 out of 30 items from the original EAQ, preserving its six-factor structure. The final factor loadings ranged from 0.39 to 0.87, with all fit indices meeting acceptable criteria. Most of the AVE and CR values exceeded 0.5 and 0.6, respectively, indicating acceptable convergent validity (Table 3) (24). As shown in Table 4, the square roots of the AVE for each factor were greater than the inter-factors correlations except for verbal abuse (0.593) and emotional rejection (0.729), which were lower than the inter-factor correlations. However, discriminant validity was still supported, confirming the scale’s divergent validity.

### Internal Consistency

The EAQ-M demonstrated strong internal consistency, with a Cronbach’s alpha of 0.901 for the full 30-item scale, which improved to 0.902 after removing item F1 (Table 5). The Cronbach’s alpha for the Terrorising subscale increased from 0.644 to 0.648, while the subscale values ranged from 0.638 to 0.81. For comparison, the original EAQ reported a Cronbach’s alpha of 0.93.

### Test-Retest Stability Using Intraclass Correlation (ICC)

For the test-retest reliability, the single measure ICC was 0.233 (95% CI: 0.155–0.363) indicating poor reliability at the individual item level, despite being statistically significant (*P* < 0.001). In contrast, the average measure ICC was 0.948 (95% CI: 0.917–0.972), reflecting excellent reliability and strong consistency over time when responses were averaged across items. These results suggest that while individual item responses may vary between measurements, the overall scale shows excellent stability in average scores across items.

## Discussion

This study translated and adapted the EAQ into Malay, ensuring its linguistic and psychometric validity. The EAQ-M, with 29 of 30 original items, is a reliable tool for assessing emotional abuse among Malaysian children. The use of an online platform minimised missing data and achieved a 100% response rate due to its flexibility, anonymity, and clarity of the questionnaire, as reflected by the strong FVI and CVI scores.

For construct validity, the model fit of EAQ-M was primarily supported by NC (< 3.0), SRMR and RMSEA, all of which were below the 0.08 cut-off. While the GFI, AGFI, CFI, TLI, and NFI values were approaching the 0.90 threshold, the GFI (0.819) and AGFI (0.782) remained slightly below it. These indices are known to be influenced by sample size, with smaller samples often yielding lower values. Although not all subscales meet the established cut-off points, previous studies have supported the model’s overall acceptability. Seçer et al. reported that GFI and AGFI values above 0.85 are acceptable (25), and other studies have suggested AGFI and TLI values above 0.80 as acceptable for CFA model fit (26, 27). Albright and Park further suggested that the CFA model fit may be deemed acceptable if three out of five fit indices meet the required standards (28). Therefore, despite some fit indices falling slightly short of ideal levels, the EAQ-M demonstrated overall robustness and an acceptable fit.

Item F1 had a low factor loading of 0.30, and its removal from the subscale further enhanced the model’s psychometric properties by improving the model fit and strengthening both internal consistency and construct validity (29). Some AVE values fell below 0.50 for Verbal Abuse (0.352), Insufficient Control (0.383), and Over Expectation (0.492), suggesting low convergent validity. This indicates that these items may have captured information from other factors rather than exclusively measuring their intended construct. This may have resulted from a small sample size, varying perceptions of emotional abuse, or challenges in translating complex emotional experiences into measurable items. The lower square roots of the AVE for Verbal Abuse (0.593) and Emotional Rejection (0.729) may reflect construct overlap, as verbal abuse often includes elements of emotional rejection. This likely represents real-world relationships rather than measurement flaws. Additionally, the shared experiences of multiple forms of abuse among participants may have increased the factor correlations.

Cronbach’s alpha was calculated for both the original 30-item and revised 29-item scales. The 29-item scale showed a slight improvement in Cronbach’s alpha, from 0.901 to 0.902, demonstrating the positive impact of removing F1 on reliability. Including both calculations of Cronbach’s alpha ensures transparency and allows for a comparison of the scale’s performance before and after modifications, providing clarity on the rationale behind the adjustments.

Two subscales of the EAQ-M, Over Expectation (0.638) and Terrorising (0.648), raised concerns regarding internal consistency as their Cronbach’s alpha scores fell below the recommended threshold of 0.7 for the established scale. These low values may reflect the challenges in capturing the complexity of emotional abuse across various contexts, potentially due to the limited number of items and item heterogeneity within these subscales (30).

To enhance the EAQ-M, cross-validation with an independent sample can confirm its robustness (31). Item response theory analysis can help refine item discrimination (32), while qualitative validation via focus groups or interviews could clarify item interpretation and the reason behind F1’s ineffectiveness (33). Longitudinal studies are also recommended to assess the revised scale’s stability and predictive validity over time. Although removing F1 optimises the psychometric properties, ongoing evaluation remains essential to ensure the long-term effectiveness of the EAQ-M (34).

The EAQ-M is a reliable tool for identifying emotional abuse among Malaysian children. Clinically, it aids early intervention by healthcare professionals (35). In schools, it helps to track emotional well-being and implement interventions (35). It also informs policies, prevention programmes, and resource allocation to protect at-risk children (36, 37).

### Limitations

The validation of EAQ-M is limited by its single-site design in Kota Bharu, which restricts generalizability. Expanding the study to include more diverse schools across Malaysia could enhance representativeness (38, 39). The relatively small sample size (*n* = 165) may have impacted parameter stability and model fit indices (e.g., GFI and AGFI), warranting larger samples for reliability. The predominantly Malay composition of the sample further limits its applicability to other ethnic groups. Future studies should consider cultural adaptations to improve relevance across Malaysia’s multiethnic population (40).

## Conclusion

The EAQ-M is a reliable and valid tool for assessing emotional abuse among Malaysian children and adolescents. With 29 items, it demonstrates excellent reliability, validity, and stability over time, making it valuable for research and clinical application. Future research should include multi-site studies across various regions in Malaysia to enhance the scale’s validity and confirm its generalizability and factor structure in more diverse populations. Revisions to subscales with low reliability and items with weak factor loadings, along with broader feedback, could further enhance its effectiveness in capturing emotional abuse across various cultural contexts.

## Figures and Tables

**Table 1:** Demographic and socioeconomic characteristics of participants (*n* = 165)

Variable	Mean (SD)	Median (IQR)	*n* (%)
Age (years)^a^	15.00 (0.85)		
Sex			
Male			60 (36.2)
Female			105 (63.6)
Race			
Malay			165 (100.0)
Chinese			0 (0.0)
Indian			0 (0.0)
Others			0 (0.0)
Number of siblings			
0–2			33 (20.0)
3–5			97 (58.8)
6–8			35 (21.2)
Place of residence			
Urban			150 (90.9)
Rural			15 (9.1)
Income (RM)^b^		2,350 (1,000–5000)	

a Age is presented as mean (SD);

b Income is presented as median and interquartile range (IQR: 25th and 75th percentile) due to right skewness;

* All other variables are presented as *n* (%)

**Table 2:** Model fit indices for initial and respecified models (Models 1–4)

	Name of index	Level of acceptance	Model 1	Model 2	Model 3	Model 4 (final model)
Parsimonious fit	*χ*^2^/df (NC)	< 3.0	1.618	1.632	1.596	1.555
Absolute fit	*χ* ^2^	*P* > 0.05	< 0.001	< 0.001	< 0.001	< 0.001
	RMSEA	< 0.08	0.061	0.062	0.060	0.058
	GFI	> 0.90	0.806	0.810	0.815	0.819
Incremental fit	AGFI	> 0.90	0.769	0.772	0.777	0.782
	CFI	> 0.90	0.849	0.854	0.863	0.873
	TLI	> 0.90	0.832	0.837	0.846	0.857
	NFI	> 0.90	0.689	0.701	0.709	0.717
Others	SRMR	< 0.08	0.085	0.086	0.084	0.081
	AIC	Lower	780.930	736.890	724.000	709.870
	BIC	Lower	1,013.880	963.620	953.840	942.820

Model 1 = initial model with 30 items, six dimensions; Model 2 = respecification of Model 1 with Item F1 removed due to low factor loading; Model 3 = Respecification of Model 2 with correlated error term added between error variance of C1 and C3; Model 4 = Respecification of Model 3 with correlated error term added between error variance of E2 and E3; NC = normed chi-square; RMSEA = root mean square error of approximation; GFI = goodness-of-fit; AGFI = adjusted goodness-of-fit; CFI = comparative fit index; TLI = Tucker-Lewis index; NFI = normed fit index; SRMR = standardised root mean square residual; AIC = Akaike information criterion; BIC = Bayesian information criterion

**Table 3:** Confirmatory factor analysis results: factor loadings, composite reliability, and average variance extracted after removal of Item F1 in the respecified final model

Factor	Item	FL	CR	AVE
Verbal abuse	A1	0.54	0.809	0.352
	A2	0.44		
	A3	0.50		
	A4	0.59		
	A5	0.52		
	A6	0.73		
	A7	0.72		
	A8	0.64		
Emotional rejection	B1	0.61	0.770	0.531
	B2	0.74		
	B2	0.82		
Overcontrol	C1	0.46	0.345	0.771
	C2	0.43		
	C3	0.30		
	C4	0.50		
	C5	0.71		
	C6	0.74		
	C7	0.79		
Insufficient control	D1	0.83	0.743	0.383
	D2	0.36		
	D3	0.70		
	D4	0.52		
	D5	0.58		
Over expectation	E1	0.66	0.741	0.492
	E2	0.81		
	E3	0.62		
Terrorising	F2	0.39	0.745	0.516
	F3	0.80		
	F4	0.87		

FL = factor loading; CR = composite reliability; AVE = average variance extracted

**Table 4:** The square root of AVE and inter-factor correlation as evidence of discriminant validity (the Fronell-Larcker criterion)

	Verbal abuse	Emotional rejection	Over control	Insufficient control	Over expectation	Terrorising
Verbal abuse	0.593*					
Emotional rejection	0.798	0.729*				
Over control	0.618	0.521	0.878*			
Insufficient control	0.707	0.796	0.542	0.619*		
Over expectation	0.488	0.548	0.486	0.588	0.701*	
Terrorising	0.277	0.298	0.307	0.254	0.260	0.718*

* The square root of AVE

**Table 5:** Comparison of Cronbach’s alpha values for the EAQ-M and original EAQ: subscale reliability and the effect on overall Cronbach’s alpha after removing F1

Factors	Number of items	Internal consistency (EAQ-M)	Internal consistency EAQ-M after removing F1	Internal consistency of the original EAQ scale
Verbal abuse	8	0.810	–	0.87
Emotional rejection	3	0.758	–	0.81
Over control	7	0.775	–	0.83
Insufficient control	5	0.733	–	0.75
Over expectation	3	0.638	–	0.77
Terrorising	4	0.644	0.648	0.84
aFull scale	30	0.901		c0.93
bAfter removing F1	29		0.902	

a Refers to the full scale of the EAQ-M with 30 items;

b Represents the Cronbach’s alpha for the EAQ-M after the removal of F1, resulting in 29 items;

c Indicates the Cronbach’s alpha values for the original EAQ, which comprises 30 items

## Appendix 1

### The Malay Version of the Emotional Abuse Questionnaire (EAQ)

This table displays the Malay version of the Emotional Abuse Questionnaire (EAQ) that resulted from a comprehensive translation process, ensuring cultural relevance and linguistic accuracy. Each item reflects careful consideration of both the original content and the target audience’s context.

**Table:**

Item label	Item description
A1	Those around me get in a fight with me *Orang sekeliling sering bergaduh atau bertengkar dengan saya*
A2	Those around me have rebuked me while talking to me or communicating with me since childhood *Orang di sekeliling saya sering menegur atau mengkritik saya setiap kali berkomunikasi dengan saya sejak kecil*
A3	Those around me did not or do not take my words seriously *Orang sekeliling tidak pernah mengambil kata-kata saya dengan serius*
A4	When I make mistake, I get blamed *Saya sering dipersalahkan apabila melakukan kesilapan*
A5	Since my childhood, I have been treated in such a way that I feel devalued *Sejak kecil saya diperlekehkan sehingga saya merasa rendah diri*
A6	Those around me used to call me bad names or they still do *Orang sekeliling memanggil saya dengan nama yang tidak baik sehingga sekarang*
A7	Those around me ridiculed or still ridicule my comments and suggestions into consideration *Orang di sekeliling saya mengejek atau memperlekehkan komen dan cadangan saya dari dulu hingga sekarang*
A8	Whatever I do, people nag at me *Orang sekeliling akan mengejek apa-apa sahaja yang saya lakukan*
B1	Those around me did not or do not take my comments and suggestions into considerations *Orang sekeliling tidak mempedulikan komen dan cadangan saya*
B2	Those around me are being cold to me *Orang di sekeliling saya bersikap dingin dan tidak menghiraukan saya*
B3	Those around me reject my feelings toward them *Orang sekeliling tidak mempedulikan perasaan saya*
C1	My parents or one of my family members used to forbid me from having relationship with my friends or they still do *Ibu bapa (atau salah seorang) atau ahli keluarga pernah atau masih melarang saya bergaul dengan rakan-rakan*
C2	Those around me used to ban me from participating in social groups or they still do (sports clubs, art clubs, etc.) *Orang sekeliling pernah atau masih melarang saya menyertai kumpulan sosial (contoh: kelab sukan, kelab seni dan lain-lain)*
C3	Those around me believe I should not have a relationship with anyone except my relatives *Orang sekeliling menganggap bahawa saya tidak sepatutnya menjalinkan hubungan dengan sesiapa kecuali keluarga sahaja*
C4	Those around me used to strongly take me under their control or they still do *Orang sekeliling mengongkong saya sejak dahulu hingga sekarang*
C5	Those around me did not or do not allow me to decide about my daily chores *Orang sekeliling tidak pernah membenarkan saya membuat keputusan tentang tugas harian saya*
C6	Those around me did not or do not allow me to decide about my field of study *Orang sekeliling tidak pernah membenarkan saya membuat keputusan untuk bidang pengajian saya*
C7	Those around me did not or do not allow me to choose my personal things based on my own style *Orang sekeliling tidak pernah membenarkan saya menguruskan hal peribadi saya mengikut cita rasa dan gaya saya sendiri*
D1	Those around me did not or do not care about what I did or what I do *Orang sekeliling tidak pernah peduli tentang apa-apa yang saya lakukan*
D2	Those around me did not or do not have any control or supervision on my relationship or what I do *Orang sekeliling tidak pernah mengawal atau melarang tentang hubungan sosial saya*
D3	Those around me kept or keep themselves busy and I was not or am not one of their concerns *Orang sekeliling tidak pernah mengambil berat atau bimbang tentang saya*
D4	The behaviours of those around me are unpredictable *Tingkah laku orang sekeliling saya sukar diramalkan*
D5	Those around me are sometimes very obsessive about what I do, whereas other times do not pay attention to me at all: their twofold behaviours have left me confused *Orang sekeliling saya kadang-kadang sangat taksub dengan apa-apa/perkara yang saya lakukan, dan pada masa lain langsung tidak menghiraukan saya*
E1	Those around me used to expect me to gain results beyond my abilities in my studies or they still do *Orang sekeliling mengharapkan saya mencapai keputusan yang melebihi kemampuan saya dalam pelajaran*
E2	Those around me used to expect me to behave beyond my potential or they still do *Orang sekeliling mengharapkan saya berkelakuan melebihi kemampuan saya sehingga sekarang*
E3	I have a feeling that I cannot full fill my family’s expectation *Saya merasa tidak dapat memenuhi harapan keluarga saya*
F1	Since childhood I was forced to do things that are not acceptable by society (like buying cigarettes) *Sejak kecil, saya dipaksa melakukan perkara yang tidak diterima masyarakat (contoh: membeli rokok)*
F2	Since childhood, I was forced to do things I did not want to, otherwise those around me threatened me to reveal my weak points in front of others. *Sejak kecil, saya dipaksa melakukan perkara yang saya tidak mahu. Jika saya menolak, orang sekeliling mengancam untuk mendedahkan kelemahan saya di hadapan orang lain*
F3	Those around me have led me to do immoral things since childhood. *Orang sekeliling telah mendorong saya melakukan perkara tidak bermoral sejak kecil*
F4	Those around me have led me to mischief since childhood *Orang sekeliling telah mendorong saya melakukan perkara terlarang sejak kecil*

A = Verbal Abuse; B = Emotional Rejection; C = Over Control; D = Insufficient Control; E = Over Expectation; F = Terrorizing

## Appendix 2

### Content Validity Index (CVI) for the Emotional Abuse Questionnaire-Malay (EAQ-M)

**Table:**

Item	Expert 1	Expert 2	Expert 3	Expert 4	Expert 5	Expert 6	Expert in agreement	I-CVI	UA
Q1	3	4	4	4	3	4	6	1	1
Q2	4	4	4	4	3	4	6	1	1
Q3	4	4	4	4	4	4	6	1	1
Q4	4	4	3	2	3	4	5	0.83	0
Q5	4	4	4	4	4	4	6	1	1
Q6	3	4	4	3	3	4	6	1	1
Q7	4	4	4	4	4	4	6	1	1
Q8	4	4	4	4	4	4	6	1	1
Q9	4	4	4	4	4	4	6	1	1
Q10	4	4	4	4	4	4	6	1	1
Q11	4	4	4	4	4	4	6	1	1
Q12	4	4	4	4	3	3	6	1	1
Q13	4	4	4	4	3	3	6	1	1
Q14	4	4	4	4	3	4	6	1	1
Q15	4	4	4	4	3	4	6	1	1
Q16	4	4	4	4	4	4	6	1	1
Q17	4	4	4	4	4	4	6	1	1
Q18	4	4	4	4	4	4	6	1	1
Q19	4	4	4	4	4	4	6	1	1
Q20	4	4	4	3	3	4	6	1	1
Q21	4	4	4	4	4	4	6	1	1
Q22	4	4	4	4	4	4	6	1	1
Q23	4	4	4	4	4	4	6	1	1
Q24	4	4	4	3	4	4	6	1	1
Q25	4	4	4	4	3	3	6	1	1
Q26	4	4	4	4	4	4	6	1	1
Q27	4	4	4	4	4	4	6	1	1
Q28	4	4	4	4	4	4	6	1	1
Q29	4	4	4	4	4	4	6	1	1
Q30	4	4	4	4	3	4	6	1	1
Proportion relevance	1	1	1	0.96	1	1	S-CVI/Ave	0.99	

							S-CVI/UA	0.96	

## Appendix 3

### Face Validity Index for the Emotional Abuse Questionnaire-Malay (EAQ-M)

**Table:**

Item	Rater 1	Rater 2	Rater 3	Rater 4	Rater 5	Rater 6	Rater 7	Rater 8	Rater 9	Rater 10	Expert in agreement	I-FVI	UA
Q1	4	4	4	4	4	4	4	4	4	4	10	1	1
Q2	4	4	4	4	4	4	4	4	4	4	10	1	1
Q3	4	4	4	4	4	4	4	4	4	4	10	1	1
Q4	4	4	4	4	4	4	4	4	4	4	10	1	1
Q5	4	4	4	4	4	4	4	4	4	4	10	1	1
Q6	4	4	4	4	4	4	4	4	4	4	10	1	1
Q7	4	4	4	4	4	4	4	4	4	4	10	1	1
Q8	4	4	4	4	4	4	4	4	4	4	10	1	1
Q9	4	4	4	4	4	4	4	4	4	4	10	1	1
Q10	4	4	4	4	4	4	4	4	4	4	10	1	1
Q11	4	4	4	4	4	4	4	4	4	4	10	1	1
Q12	4	4	4	4	4	4	4	4	4	4	10	1	1
Q13	4	4	4	4	4	4	4	4	4	4	10	1	1
Q14	4	4	4	4	4	4	4	4	4	4	10	1	1
Q15	4	4	4	4	4	4	4	4	4	4	10	1	1
Q16	4	4	4	4	4	4	4	4	4	4	10	1	1
Q17	4	4	4	4	4	4	4	4	4	4	10	1	1
Q18	4	4	4	4	4	4	4	4	4	4	10	1	1
Q19	4	4	4	4	4	4	4	4	4	4	10	1	1
Q20	4	4	4	4	4	4	4	4	4	4	10	1	1
Q21	4	4	4	4	4	4	4	4	4	4	10	1	1
Q22	4	4	4	4	4	4	4	4	4	4	10	1	1
Q23	4	4	4	4	4	4	4	4	4	4	10	1	1
Q24	4	4	4	4	4	4	4	4	4	4	10	1	1
Q25	4	4	4	4	4	4	4	4	4	4	10	1	1
Q26	4	4	4	4	4	4	4	4	4	4	10	1	1
Q27	4	4	4	4	4	4	4	4	4	4	10	1	1
Q28	4	4	4	4	4	4	4	4	4	4	10	1	1
Q29	4	4	4	4	4	4	4	4	4	4	10	1	1
Q30	4	4	4	4	4	4	4	4	4	4	10	1	1
Proportion relevance	1	1	1	1	1	1	1	1	1	1	S-FVI/Ave	1	

											S-FVI/UA	1	
